# Prediction of Thiol Group Changes in Minced Raw and Cooked Chicken Meat with Plant Extracts—Kinetic and Neural Network Approaches

**DOI:** 10.3390/ani11061647

**Published:** 2021-06-01

**Authors:** Anna Kaczmarek, Małgorzata Muzolf-Panek

**Affiliations:** Department of Food Quality and Safety Management, Faculty of Food Science and Nutrition, Poznań University of Life Sciences, Wojska Polskiego 31, 60-637 Poznań, Poland; malgorzata.muzolf-panek@up.poznan.pl

**Keywords:** thiol content, protein oxidation, raw chicken, cooked chicken, plant extracts, predictive models, temperature effect, Arrhenius equation, artificial neural network

## Abstract

**Simple Summary:**

Demand for poultry meat (chickens and turkeys) is constantly increasing. The upward trend in the production and consumption of poultry meat has two reasons. The first is the financial aspect because chicken meat is relatively cheap. The second reason is the nutritional and health aspect. Although the meat has high nutritional, dietary, culinary, technological, and sensory values, it is very susceptible to undesirable changes during storage, mainly due to the growth of microflora but also due to lipid and protein oxidation. The use of plant extracts in food technology is multifunctional, as they exhibit antioxidant and antibacterial effects and have a beneficial effect on the texture of meat and meat products. Moreover, the antioxidant effect of compounds isolated from plants may influence consumer health. Antioxidants of plant origin can be used as an additive to animal feed, as well as a component of stuffing or marinating mixes for meat. In addition, they are used in the coating of raw materials or in active packaging for food products. So far, many studies have shown the positive effect of plant and plant extract addition to meat on the oxidative status of its protein. However, the predictive approach to protein oxidation in raw meat is still little described. This study has demonstrated the potential usefulness of the kinetic model as well as models based on artificial neural networks (ANNs) to the realistic prediction of protein oxidation expressed as thiol group (SH) changes in raw and cooked chicken meat during storage. Such predictive models allow us to predict oxidative changes in minced meat under different time and temperature conditions as minced meat is particularly susceptible to oxidation through exposure to oxygen during the mincing process itself and through the increased contact surface with oxygen. This knowledge is very useful in designing food products and predicting their shelf-life. Additionally, the effectiveness of various spices in the raw and cooked meat system were compared. Meat is a very complex system and, according to the research, there is no direct correlation between the anti-oxidant activity of the spice itself and its antioxidant effectiveness in the product.

**Abstract:**

The aim of the study was to develop predictive models of thiol group (SH) level changes in minced raw and heat-treated chicken meat enriched with selected plant extracts (allspice, basil, bay leaf, black seed, cardamom, caraway, cloves, garlic, nutmeg, onion, oregano, rosemary, and thyme) during storage at different temperatures. Meat samples with extract addition were stored under various temperatures (4, 8, 12, 16, and 20 °C). SH changes were measured spectrophotometrically using Ellman’s reagent. Samples stored at 12 °C were used as the external validation dataset. SH content decreased with storage time and temperature. The dependence of SH changes on temperature was adequately modeled by the Arrhenius equation with average high R^2^ coefficients for raw meat (R^2^ = 0.951) and heat-treated meat (R^2^ = 0.968). Kinetic models and artificial neural networks (ANNs) were used to build the predictive models of thiol group decay during meat storage. The obtained results demonstrate that both kinetic Arrhenius (R^2^ = 0.853 and 0.872 for raw and cooked meat, respectively) and ANN (R^2^ = 0.803) models can predict thiol group changes in raw and cooked ground chicken meat during storage.

## 1. Introduction

According to the United Nations Food and Agriculture Organization, pork is the most widely eaten meat in the world (36%) followed by poultry (33%), beef (24%), and goat/sheep (5%). Poultry meat is the second-most consumed meat in the world representing a valuable source of nutrients, such as high-quality proteins, microelements, vitamins, and polyunsaturated fatty acids (PUFAs) [1].

Oxidation of meat proteins (Pox) may reduce sensory quality by decreasing tenderness and juiciness, due to effects on proteolytic enzyme activity, causing changes in flavor and color [2]. Among the oxidative changes in meat proteins, the most important are the formation of hydroperoxides and carbonyl derivatives, loss of sulfhydryl groups, formation of protein cross-linking occurring due to the formation of disulfide bonds (so-called cross-linking), peptide fragmentation, and reduction of protein solubility [2]. This is attributed to the decrease in the activity of the peptidase calpain in meat through oxidation and myosin crosslinking. Cystine is the main crosslinked in oxidized meat products [3,4]. Protein oxidation (during mincing, storage, and heat treatment) not only leads to a deterioration of the functional properties of meat, but also contributes to a decrease in its nutritional value due to loss of essential amino acids and a decrease in digestibility [5]. Moreover, the potential health risk attributed to oxidized protein intake was recently reviewed [6,7]. Herbs and spices have been used by humans since ancient times. Nowadays, the addition of spices and herbs to food is not only connected with enriching the organoleptic properties of food products. They are a source of natural antioxidant substances which can prolong food shelf-life [8] and exhibit health-promoting effects when added to meat products [9]. The protection against protein oxidation is very complex, therefore the antioxidant activity of phenolic compounds from plant extracts cannot be directly transferred to their action in the food matrix. Polyphenols can also act prooxidatively and lead to protein, carbohydrate, and DNA damage [10]. It was found that extracts from green tea induce protein polymerization, due to the formation of covalent protein–phenol interaction [11,12]. Garlic essential oil was found also to promote protein oxidation during chill storage of pork patties [13]. It is therefore important to test the antioxidant efficacy of plant extracts both in terms of matrix and concentration. While the effects of antioxidants in isolated systems have been intensely studied and are often used for the evaluation of health-promoting effects of food containing such antioxidants, the reactivity of the same antioxidants in food systems especially under processing conditions is only beginning to be understood.

Probably the most widely used routine method for protein oxidation measurement is the determination of carbonyl groups that react with 2,4-dinitrophenylhydrazine (DNPH) to form the corresponding hydrazones, which are then quantified spectrophotometrically. Carbonyl compounds not related to protein oxidation may be formed during lipid peroxidation and the Maillard reaction leading to an overestimation of protein oxidation [14]. Another commonly used method to assess oxidative changes in meat is the determination of thiol groups in meat [14,15,16,17,18,19,20]. Free thiol groups may be analyzed by spectrophotometric after derivatization with Ellman’s reagent 86 (5,5′-dithio-bis(2-nitrobenzoic acid) (DTNB)), which leads to the formation of a mixed disulfide and release of 5-thio-2-nitrobenzoic acid. Contrarily to the DNPH method, the spectrophotometric method using Ellman’s reagent does not require solubility of the protein, but can be applied in suspension [14].

Kinetic models as well as models based on artificial neural networks (ANNs) are a powerful tool for studying the changes in food quality indices during the storage period [21,22,23,24,25,26,27,28,29,30]. Therefore, the aim of the study was to develop predictive models of protein oxidation, expressed in thiol (SH) group decrease in minced raw and heat-treated chicken meat enriched with selected plant extracts during storage at different temperatures.

## 2. Materials and Methods

### 2.1. Materials

All herbs (basil, bay leaf, oregano, rosemary, and thyme) and spices (allspice, black seed, cardamom, caraway, cloves, garlic, nutmeg, and onion) were bought at a local distributor (Ciecierzyn, Poland). Chicken legs were supplied by the local producer (Swarzędz, Poland). The meat was deboned and minced (diameter of plate = 5 mm) on the day of transport to the laboratory. The temperature during transport was held at the level of 4–8 °C. The basic composition of meat was as follows: 74.31% of moisture, 18.3% of protein, and 6.4% of fat.

### 2.2. Preparation and Characterization of Plant Extract

Extracts of spices and herbs were prepared in 50% aqueous ethanol as previously described with the ratio 1:15 (m/v) [31]. The DPPH^•^ radical scavenging capacity was determined by the method of Sánchez-Moreno et al. [32], modified as described previously [33]. Final results were expressed as µmol Trolox equivalent (TE) per g of dried plant. The content of phenolic compounds was investigated by the method of Singleton and Rossi [34] and total phenolic content (TPC) was expressed as mg GAE (gallic acid equivalent) per g of dried plant. The results were previously presented [33,35]. and discussed carefully, thus they are no longer included in the Result section, but cited below in Table 1.

### 2.3. Meat Sample Preparation and Storage Conditions

The frozen dried extract was dissolved in water (60 mL) on the day it was added to the meat (3 kg). The concentration of the spice extract expressed in g of powdered spices used for extraction per 100 g of meat was therefore 0.5% (m/m). Twenty-eight samples were prepared from raw minced chicken: one control (C) (meat without extract, only mixed with 60 mL of water) and thirteen samples, namely with allspice, basil, bay leaf, black seed, cardamom, caraway, cloves, garlic, nutmeg, onion, oregano, rosemary, and thyme 0.5% (m/m). Then, each sample was mixed separately for 3 min and placed in a low-density polyethylene bag. Half of the samples were subjected to thermal treatment in a water bath (80 °C, 30 min) while the rest were left raw, and stored at 4, 8, and 12 °C for 13 days and at 16 and 20 °C for 5 days.

### 2.4. SH Content

Protein oxidation was investigated in terms of changes in SH content which was measured spectrophotometrically (Cary 1E spectrophotometer, Varian, Belrose, Australia) using Ellman’s reagent [26] by the modified method [31]. Final results were expressed as nmol cysteine per mg of protein. In order to obtain universal models, SH changes during storage were given in percentage. The 0 day value was used as the initial value with SH equaled to 100%.

### 2.5. The Kinetic Model

Quality changes in time could be described by the general equation:

(1) $$-\mathrm{dQ}/\mathrm{dt}={\mathrm{kQ}}^{n},$$

where Q is a quality index, t is time, k is kinetic constant rate which is temperature dependent, and n is kinetic order [36].

The zero-order equation was implemented for SH changes during storage at constant temperature and the Equation (1) for n = 0 gave:

(2) $$SH=SH_{0}-kt,$$

where SH is a content of thiol groups (%), SH_0_ is the initial value (100%) at time 0, k is the meat quality rate constant (day^−1^) at a given temperature, and t is time (day). SH changes at 4, 8, 16, and 20 °C were used to establish the kinetic models. Linear regression was obtained by plotting SH changes (%) versus time (day).

Temperature-dependence of the rate constant (k) was introduced to the models by the Arrhenius equation [37]:

(3) $$k=k_{0}\;exp\left(-E_{a}/RT\right),$$

where *k* (day^−1^) represents the SH content rate, *k*_0_ is the pre-exponential factor, *Ea* (kJ/mol) is the activation energy, *R* is the universal gas constant, and *T* is absolute temperature.

The linearized form of the Arrhenius equation is:

(4) $$lnk=lnk_{0}-E_{a}/RT,$$

The slope of the plot of ln*k* on the reciprocal of *T* equaled to −*E_a_/R* and an intercept was ln*k*_0_. The Arrhenius model used for the prediction of product quality is an empirical rather than a physical one. Because of the complexity of food matrix, many reaction could have occurred at the same time, thus in food, temperature dependence is investigated for very complicated reactions and not for defined, simple reactions [38].

### 2.6. Artificial Neural Networks (ANNs)

ANNs use storage conditions (time and temperature), plant extract addition, and thermal treatment as input data for the calculation. The datasets were divided into three subsets in a ratio of 70:15:15. These was a learning set (a set of samples used to adjust the network weights), a validation set (a set of samples used to tune the parameters), and a test set (a set of samples used only to assess the performance to new, unseen observations). The Broyden–Fletcher–Goldfarb–Shanno learning algorithm (200 epoch) was used for training multilayer feed-forward-connected ANN, and multilayer perceptron (MLP) and radial basis function (RBF) networks were used to search for the appropriate ANN model. The best five networks were retained. The network architecture was as follow: one input layer (20 neurons), one hidden layer (8–10 neurons), and one output layer (1 neuron). The sums of squares and the cross-entropy error function were used during the network training process. The success of the model to predict thiol groups levels was assessed as: training performance as a percentage of the samples in the learning set correctly predicted during the networks learning step, test performance as a percentage of the samples in the testing set correctly predicted during the network testing step, and validation performance as a percentage of the samples in the validation set (samples not used in the learning and testing steps) correctly predicted by the models during the network validation step.

### 2.7. Validation and Evaluation of Kinetic and ANN Models

The external validation was performed. Thiol group change models at 4, 8, 16 and 20 °C were established by combining kinetic analysis and the Arrhenius equation as well as ANN models. SH changes at 12 °C were adopted to evaluate the performance of obtained predictive models.

### 2.8. Multiple Linier Regression Analysis (MLR)

As a predictive analysis, the multiple linear regression was used to compare the rates (slope of regression equation) of thiol group degradation in raw and cooked chicken meat samples with different plant extracts within the storage period at a given temperature. The general model of MLR has a following equation:

(5) $$y=\beta_{0}+\beta_{1}\;x_{1}+\beta_{2}\;x_{2}+\ldots +\beta_{k}x_{k}+\varepsilon ,$$

where *y* is the variable value, *β*_0_ is the intercept, *β*_1−*k*_ is the regression coefficient; *x*_1−*k*_ are the predictors, and ε is the standard estimation error. The comparisons between the coefficients were performed introducing 13 (*k* − 1) dummy variables as predictors to regression analysis. The control samples were not coded because this was the category with which all other categories would be compared. The significant differences between the regression coefficients were based on the result of the *t*-test (*p* ≤ 0.05) for dummy variables.

### 2.9. Statistical Analysis

Thiol group measurements were run in triplicate and the results were expressed as mean ± standard deviations (SD). The statistical tests were performed using Statistica 13.3 software (StatSoft, Tulsa, Oklahoma, USA). A significance level of *p* = 0.05 was used.

Values of kinetic parameters were evaluated using non-linear estimation analysis by least-squares criterion with Levenberg–Marquardt algorithm. The goodness of fit of the models was verified based on the determination coefficient (R^2^) and root-mean-square error (RMSE).

## 3. Results and Discussion

### 3.1. Development of Kinetic Models for Thiol Groups Decrease in Ground Chicken Meat

All meat samples were kept under controlled conditions and taken for analysis at appropriate time intervals to allow efficient kinetic analysis of SH group degradation. Thiol group loss is one of the indices providing information on the extent of protein oxidation and it was reported that free thiols were highly correlated with the other markers such as the content of carbonyl compounds [10,39]. This method has been successfully applied in raw meat chicken meat [31], cooked chicken meat [11], and dried chicken meat [15]. The highest regression coefficients values were obtained for the plot of SH values vs. time. Therefore, the zero-order reaction model was applied (Equation 2). The same reaction order was observed in SH group decay in rabbit meat [40]. The effect of temperature was included to the mathematical models using the Arrhenius law (Equation 3). The predictive models were obtained by integrating Equations (2) and (3).

The obtained predictive equations are presented in Table 2.

High coefficients of determination were recorded for the parameters of the models for both raw meat (average R^2^ = 0.951) and heat-treated meat (R^2^ = 0.968). The highest R^2^ values were obtained for basil samples for raw chicken meat (above 0.998), whereas the lowest was for onion (R^2^ = 0.87). Among cooked meat samples the highest value of determination coefficient was reported for control sample (R^2^ = 0.998) whereas the lowest was for allspice-treated samples. All model parameters are shown in Table 2. The activation energy E_a_ can be seen as the energy barrier that molecules need to cross in order to be able to react. The proportion of molecules able to do that increases with temperature, which qualitatively explains the effect of temperature on rates. Since the SH content was monitored in the meat system the concept of Ea as the minimum energy required for the reaction should be discussed very carefully, which was mentioned by van Boekel [38]. In this study Ea values indicated how sensitive to temperature the samples were. This suggested that protein oxidation in raw chicken meat was the most sensitive to temperature in the caraway-treated sample (E_a_ = 116.1 kJ/mol), whereas it was the least sensitive to temperature in the control sample (E_a_ = 33.05 kJ/mol). Therefore, the samples can be ordered from the least sensitive to temperature to the most sensitive in the following order: control < onion < oregano < garlic < rosemary < basil < bay leaf < thyme < black seed < clove < nutmeg < cardamom < allspice < caraway. Meanwhile, Ea values for SH groups decreased in heat-treated chicken meat samples and varied from 25.01 kJ/mol for garlic-treated samples to 90 kJ/mol for caraway-treated samples. Therefore, the samples can be ordered from the least sensitive to temperature to the most sensitive in the following order: garlic < onion < thyme < control < cardamom < basil < allspice < rosemary < black seed < nutmeg < bay leaf < caraway. On the basis of the Arrhenius equation parameters, it can be concluded that protein oxidation, expressed as a decrease in thiol groups, is the most temperature-dependent in meat samples with added caraway.

The physical meaning of k_0_ is that it represents the rate constant at which all molecules have sufficient energy to react (Ea = 0). The highest k_0_ values were noted for caraway-treated raw (k_0_ = 1.19 × 10^22^) as well as heat-treated meat samples. While the lowest k_0_ values were 1.2 × 10^7^ and 4.19 × 10^5^ for the control in raw meat and garlic-treated cooked meat, respectively.

The goodness of fit of Arrhenius models is presented in Table 3. The average value of adjusted R^2^ coefficient for observed and predicted SH values was 0.923 and 0.952 for raw and cooked chicken meat, respectively. The highest sum of R^2^ was noted for the basil-treated (3.94) raw meat sample and the cardamom-treated (3.94) cooked meat sample, whereas the lowest values of this statistic were observed for nutmeg addition (3.21) to raw meat and thyme addition (3.54) to cooked chicken meat. The Arrhenius models for cooked meat samples showed slightly better goodness of fit than the Arrhenius models obtained for raw meat samples with average sum of R^2^ values of 3.8 and 3.7, respectively. The better fit of the predictive models of the changes in thiol group content in cooked meat samples than in raw meat was also evidenced by the RMSE values which reached average values of 4.38 and 5.55, respectively. The obtained results demonstrated that kinetic models could accurately predict changes in the content of thiol groups in raw and heat-treated meat samples enriched with plant extracts under various time–temperature conditions.

### 3.2. ANN Models for Thiol Groups Decrease in Ground Chicken Meat

Data for all samples (with and without plant extracts) were used to construct ANNs. The best five ANN–MLP networks are presented in Table 4. In neural networks obtained for SH values the tanh, exponential, and logistic functions were used in the hidden layer, while tanh and linear functions were used in the output layer. The number of neurons in the hidden layer varied from 12 to 29. The goodness of fit of all selected networks was very high with R^2^ above 0.96 for all networks. The best network was MLP 20-19-1 with the highest adjusted determination coefficient (R^2^ = 0.983) and the lowest RMSE (10.13) values.

### 3.3. Validation and Evaluation of SH Prediction Models

**Figure 1:**
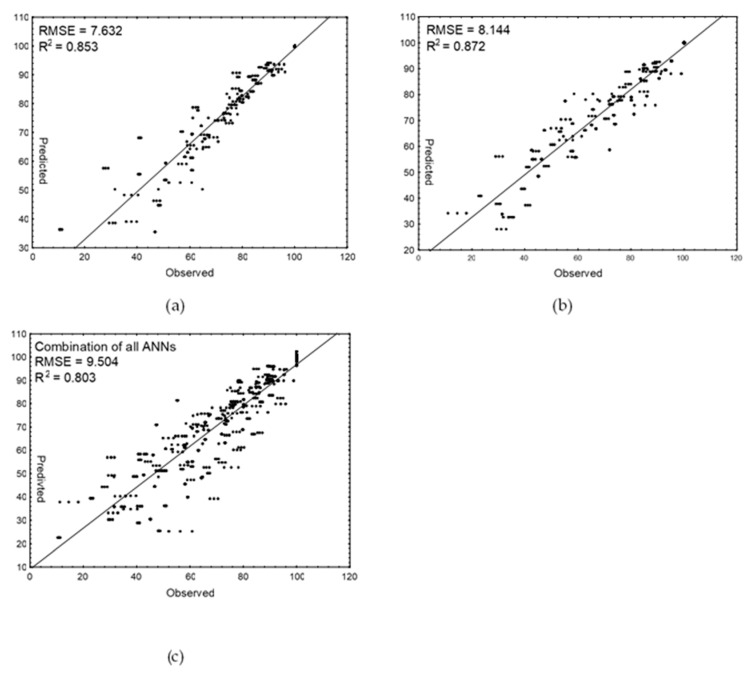
Predictability of the Arrhenius models for (**a**) raw chicken meat and (**b**) cooked chicken meat. (**c**) ANN model for the SH value changes in raw and cooked meat samples enriched with plant extracts stored at 12 °C. The solid line represents a perfect match between experimental and predicted values.

The external validation of obtained predictive models was performed. The validation data set consisted of measurements of thiol group levels in raw and cooked meat samples stored at 12 °C. The SH value changes during meat sample storage predicted using these three models were plotted against the observed values (Figure 1). The scatter plots revealed a high degree of linearity which was confirmed by high adjusted regression coefficients (0.8–0.87) and low RSME values. Comparing the kinetic models, a slightly better model fit to the experimental data was found for the predictive model of SH group changes in heat-treated meat (Figure 1a) than in raw meat (Figure 1b). Previously, Arrhenius and ANNs models were developed based on the changes of various indices in rabbit [40], chicken [41], beef [42], and pork [43] meat and meat products as well as in seafoods [35,44,45].

**Figure 2:**
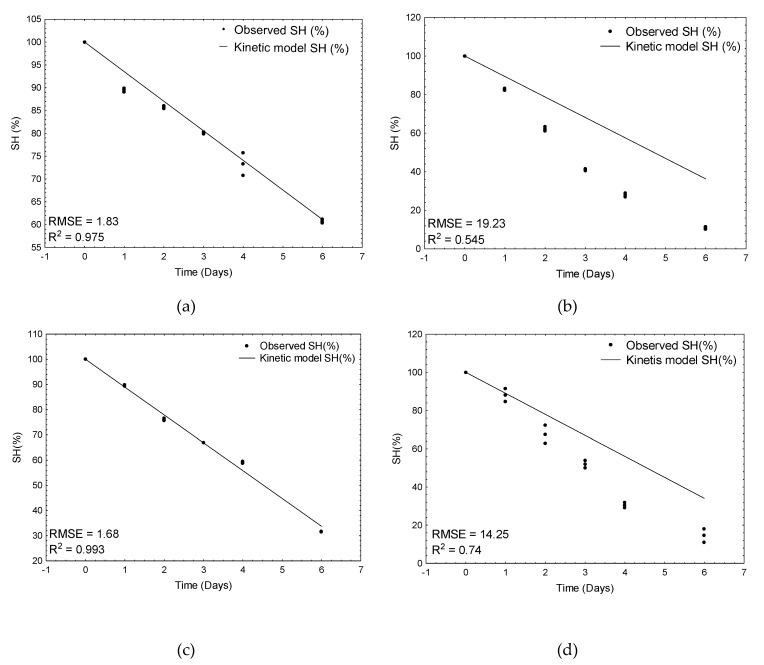
Verification of kinetic models: (**a**) the best fit for bay-leaf-treated raw meat samples, (**b**) the worst fit for control raw meat samples, (**c**) the best fit for cardamom-treated cooked meat samples, and (**d**) the worst fit for garlic-treated meat samples.

The best and the worst models for raw and cooked meat are shown in the Figure 2. As can be observed, the worst-fitted models significantly underestimated the decrease in SH groups in the control samples and in the garlic-treated meat samples of raw and cooked meat, respectively. The model based on artificial neural networks (combined of all best five networks) was characterized by the worst predictive ability with the highest RMSE value (9.5) and the lowest determination coefficient (0.803). The reason is that this model, apart from the effect of temperature (as in kinetic ones), additionally and simultaneously predicted the effects of both plant additives and thermal treatment on the decrease of SH groups in meat samples.

### 3.4. Regression Modeling Using MLR

To assess the influence of time, temperature, and addition of plant extracts on thiol group levels in raw and cooked chicken meat, multiple linear regression (MLR) was per-formed. The results of regression analysis are shown in Table 5. The models of multiple regression analysis were statistically significant with *p* < 0.05 for both the raw and cooked meat systems. The determination coefficients and standard errors were equal to 0.64 and 12.61, and 0.79 and 9.68 for raw and cooked meat, respectively. Both storage time and temperature were statistically significant in reducing the content of SH groups in both raw and cooked meat samples. According to the regression coefficient values, the best ability to inhibit the oxidation process in raw meat samples is possessed bay leaf extract, with the highest slope value (13.48). Clove extract was also very effective (slope value 12.26) which is in accordance with previously published work [31]. However, caraway extract was the most effective in heat-treated meat samples with a slope value of 13.3 and nutmeg extract showed a statistically significant antioxidant effect in both raw and cooked meat systems. The differences in antioxidant potential of plant extracts in raw and cooked meat samples demonstrated the significant effect of heat treatment of meat on the effectiveness of the plant extracts used. Some antioxidant compounds are thermolabile and are no more effective after heat treatment. In raw meat, nine of the tested extracts showed antioxidant activity and in cooked meat only five of them did. Gallo et al. [46] reported a protective action of *Echinacea angustifolia* extract against protein oxidation in chicken meat. Jongberg et al. [11] reported that mate extract reduced thiol loss in chicken meat. Oregano can also prevent protein oxidation during the cooking of chicken-meat burgers [1]. In this study, thyme extract exhibited statistically significant prooxidant effect in cooked meat samples. It was previously proven that green tea extract [12] as well as garlic extract [13] could promote protein oxidation in meat. Bay leaf was found to be the most active in inhibiting oxidative changes in raw chicken meat proteins although it showed moderated antioxidant activity (Table 1).

## 4. Conclusions

This study explores the effect of temperature and antioxidant properties of selected culinary species and herbs on protein oxidation, measured by thiol group loss, in raw and cooked minced chicken meat during storage. The experimental data of SH values were fitted to kinetic models and ANN models. The changes in SH were dependent on temperature and well described by the zero-order kinetic model. The kinetic rate constant can be modeled using the Arrhenius equation with satisfactory accuracy. To conclude, the models employed can be used for the prediction of oxidative changes in the chicken meat protein. The kinetic model for cooked meat showed better fit than the model for raw meat. The worst fit was noted for the model built using ANNs. The ANN model, apart from the effect of temperature, additionally and simultaneously predicted the effects of both plant additives and thermal treatment on the content of SH groups in meat samples. Additionally, based on the obtained parameters, the antioxidant capacity of plant extracts was compared. This research demonstrated the differences in effectiveness of plant extract in raw and heat-treated meat samples. The meat is a very complex system and, according to the research, there is no direct correlation between the antioxidant activity of the spice itself and its antioxidant effectiveness in the product.

This study showed the potential usefulness of the models for realistic prediction of the SH changes in raw and cooked chicken meat during storage. Such predictive models allow us to predict oxidative changes in minced meat under different time and temperature conditions. This knowledge is very useful in designing food products and predicting their shelf-life.

## Figures and Tables

**Table 1:** Antioxidant activity and phenolic compound content of ethanol in water (1/1v/v/) extracts.

Extracts	DPPH µM TE/g	TPC mg GAE g/DW
Allspice *	555 ± 24 ^g^	31.61 ± 0.81 ^e^
Basil	134.7 ± 2.3 ^c^	14.81 ± 0.35 ^bc^
Bay leaf *	231.9 ± 1.5 ^e^	22.56 ± 0.16 ^cd^
Black seed *	7.59 ± 0.84 ^a^	2.46 ± 0.61 ^a^
Cardamom *	5.45 ± 0.35 ^a^	1.24 ± 0.01 ^a^
Caraway *	20.2 ± 0.6 ^a^	2.39 ± 0.14 ^a^
Clove *	1443 ± 1 ^h^	167.2 ± 9.3 ^f^
Garlic	14.8 ± 1.6 ^a^	3.6 ± 0.05 ^a^
Nutmeg *	22.22 ± 0.15 ^ab^	3.89 ± 0.14 ^a^
Onion	5.74 ± 0.28 ^a^	7.05 ± 0.58 ^ab^
Oregano	171.6 ± 5.8 ^d^	20.7 ± 0.1 ^cd^
Rosemary	50.4 ± 3.6 ^b^	4.66 ± 0.36 ^a^
Thyme	278.3 ± 16.2 ^f^	23.5 ± 0.6 ^d^

All values are mean ± SD of the three replicates [33]. * data from TPC, total polyphenol content. ^a–h^, means with the same superscript within the same column are not different (*p* > 0.05).

**Table 2:** Kinetic models for SH values changes of ground chicken with plant extracts during storage at various temperatures.

Plant Extract	Raw Chicken Meat		Cooked Chicken Meat	
	Equation	R^2^	Equation	R^2^
Control	$SH=SH_{0}-1.2\times 10^{7}\times \exp\left(-33.05/\left(R\times T\right)\right)\times t$	0.941	$SH=SH_{0}-7.98\times 10^{8}\times \exp\left(-42.49/\left(R\times T\right)\right)\times t$	0.998
Allspice	$SH=SH_{0}-1.66\times 10^{20}\times \exp\left(-105.63/\left(R\times T\right)\right)\times t$	0.905	$SH=SH_{0}-8.94\times 10^{9}\times \exp\left(-48.9/\left(R\times T\right)\right)\times t$	0.899
Basil	$SH=SH_{0}-2.46\times 10^{10}\times \exp\left(-51.1/\left(R\times T\right)\right)\times t$	0.998	$SH=SH_{0}-4.92\times 10^{9}\times \exp\left(-47.6/\left(R\times T\right)\right)\times t$	0.979
Bay leaf	$SH=SH_{0}-3.09\times 10^{12}\times \exp\left(-63.75/\left(R\times T\right)\right)\times t$	0.977	$SH=SH_{0}-3.37\times 10^{16}\times \exp\left(-85.13/\left(R\times T\right)\right)\times t$	0.985
Black seed	$SH=SH_{0}-6.34\times 10^{13}\times \exp\left(-70.5/\left(R\times T\right)\right)\times t$	0.989	$SH=SH_{0}-6.06\times 10^{11}\times \exp\left(-58.77/\left(R\times T\right)\right)\times t$	0.950
Caraway	$SH=SH_{0}-1.19\times 10^{22}\times \exp\left(-116.1/\left(R\times T\right)\right)\times t$	0.963	$SH=SH_{0}-3.34\times 10^{17}\times \exp\left(-92.00/\left(R\times T\right)\right)\times t$	0.981
Cardamon	$SH=SH_{0}-8.15\times 10^{18}\times \exp\left(-97.97/\left(R\times T\right)\right)\times t$	0.970	$SH=SH_{0}-3.85\times 10^{9}\times \exp\left(-46.63/\left(R\times T\right)\right)\times t$	0.996
Clove	$SH=SH_{0}-2.18\times 10^{17}\times \exp\left(-90.47/\left(R\times T\right)\right)\times t$	0.963	$SH=SH_{0}-3.51\times 10^{12}\times \exp\left(-63.58/\left(R\times T\right)\right)\times t$	0.979
Garlic	$SH=SH_{0}-1.65\times 10^{9}\times \exp\left(-45.12/\left(R\times T\right)\right)\times t$	0.882	$SH=SH_{0}-4.19\times 10^{5}\times \exp\left(-25.01/\left(R\times T\right)\right)\times t$	0.926
Nutmeg	$SH=SH_{0}-3.49\times 10^{17}\times \exp\left(-90.86/\left(R\times T\right)\right)\times t$	0.914	$SH=SH_{0}-4.2\times 10^{15}\times \exp\left(-80.68/\left(R\times T\right)\right)\times t$	0.998
Onion	$SH=SH_{0}-1.55\times 10^{8}\times \exp\left(-39.2/\left(R\times T\right)\right)\times t$	0.870	$SH=SH_{0}-5.09\times 10^{7}\times \exp\left(-37.34/\left(R\times T\right)\right)\times t$	0.934
Oregano	$SH=SH_{0}-1.04\times 10^{9}\times \exp\left(-43.72/\left(R\times T\right)\right)\times t$	0.961	$SH=SH_{0}-1.49\times 10^{9}\times \exp\left(-44.52/\left(R\times T\right)\right)\times t$	0.995
Rosemary	$SH=SH_{0}-1.8\times 10^{10}\times \exp\left(-50.87/\left(R\times T\right)\right)\times t$	0.988	$SH=SH_{0}-1.17\times 10^{10}\times \exp\left(-49.22/\left(R\times T\right)\right)\times t$	0.991
Thyme	$SH=SH_{0}-2.76\times 10^{13}\times \exp\left(-68.36/\left(R\times T\right)\right)\times t$	0.996	$SH=SH_{0}-1.67\times 10^{8}\times \exp\left(-38.99/\left(R\times T\right)\right)\times t$	0.945

**Table 3:** The goodness of fit of Arrhenius models of thiol group changes in ground chicken meat with various plant extract additions during storage at different temperatures.

Extract	(K)	Arrhenius Model					
		Raw Chicken Meat			Cooked Chicken Meat		
		R^2^	RMSE	ΣR^2^	R^2^	RMSE	ΣR^2^
Control	277	0.9243 ± 0.0587	5.75 ± 0.30	3.81	0.9947 ± 0.0044	1.64 ± 0.55	3.99
	281	0.9636 ± 0.0290	4.26 ± 0.21		0.9945 ± 0.0042	1.64 ± 0.11	
	289	0.9309 ± 0.0552	6.17 ± 0.50		0.9979 ± 0.0016	1.20 ± 0.15	
	293	0.9931 ± 0.0051	2.26 ± 0.26		0.9987 ± 0.0011	1.13 ± 0.25	
Allspice	277	0.9942 ± 0.0043	1.86 ± 0.16	3.66	0.9800 ± 0.0157	2.84 ± 0.35	3.64
	281	0.8000 ± 0.1693	7.21 ± 0.76		0.7987 ± 0.1614	9.73 ± 0.59	
	289	0.9258 ± 0.0526	8.30 ± 0.45		0.9192 ± 0.0632	7.19 ± 0.53	
	293	0.9433 ± 0.0309	10.22 ± 1.45		0.9461 ± 0.0394	6.72 ± 0.04	
Basil	277	0.9082 ± 0.0739	5.14 ± 0.54	3.74	0.8782 ± 0.0986	5.72 ± 0.37	3.75
	281	0.9236 ± 0.0606	5.59 ± 0.16		0.8936 ± 0.0867	5.72 ± 0.80	
	289	0.9984 ± 0.0009	1.21 ± 0.53		0.9886 ± 0.0092	2.52 ± 0.69	
	293	0.9146 ± 0.0640	9.57 ± 1.30		0.9893 ± 0.0075	3.27 ± 0.05	
Bay Leaf	277	0.9946 ± 0.0056	1.04 ± 0.44	3.94	0.9662 ± 0.0269	3.69 ± 0.21	3.80
	281	0.9768 ± 0.0200	1.94 ± 0.12		0.9760 ± 0.0209	2.24 ± 0.32	
	289	0.9813 ± 0.0155	2.38 ± 0.06		0.9468 ± 0.0405	6.55 ± 0.22	
	293	0.9921 ± 0.0061	2.34 ± 0.15		0.9063 ± 0.0553	12.70 ± 1.61	
Black seed	277	0.9902 ± 0.0082	1.75 ± 0.36	3.83	0.9923 ± 0.0059	1.98 ± 0.42	3.83
	281	0.8937 ± 0.0893	4.41 ± 0.07		0.9349 ± 0.0543	5.07 ± 0.66	
	289	0.9919 ± 0.0075	1.77 ± 0.51		0.9248 ± 0.0556	7.92 ± 0.37	
	293	0.9584 ± 0.0309	6.19 ± 0.10		0.9804 ± 0.0129	4.87 ± 0.09	
Caraway	277	0.9987 ± 0.0010	0.97 ± 0.40	3.59	0.9978 ± 0.0018	0.62 ± 0.15	3.94
	281	0.9700 ± 0.0260	2.76 ± 0.37		0.9493 ± 0.0510	1.76 ± 0.83	
	289	0.7347 ± 0.1785	18.35 ± 0.13		0.9965 ± 0.0030	0.93 ± 0.22	
	293	0.8856 ± 0.0758	12.92 ± 0.40		0.9956 ± 0.0033	2.01 ± 0.38	
Cardamom	277	0.9980 ± 0.0014	1.29 ± 0.48	3.63	0.9972 ± 0.0030	0.89 ± 0.26	3.96
	281	0.8114 ± 0.1521	7.88 ± 0.43		0.9938 ± 0.0065	1.30 ± 0.66	
	289	0.9056 ± 0.0570	11.72 ± 0.22		0.9751 ± 0.0186	4.12 ± 0.08	
	293	0.9177 ± 0.0441	12.70 ± 0.20		0.9967 ± 0.0025	1.82 ± 0.23	
Clove	277	0.9988 ± 0.0008	0.67 ± 0.30	3.87	0.9687 ± 0.0348	2.51 ± 1.50	3.79
	281	0.9958 ± 0.0046	1.09 ± 0.65		0.9711 ± 0.0235	2.87 ± 0.29	
	289	0.9139 ± 0.0675	7.13 ± 0.24		0.9512 ± 0.0395	4.62 ± 0.23	
	293	0.9651 ± 0.0252	5.94 ± 0.26		0.9032 ± 0.0638	11.30 ± 0.31	
Garlic	277	0.9510 ± 0.0405	3.70 ± 0.94	3.73	0.9635 ± 0.0303	3.93 ± 0.30	3.78
	281	0.9053 ± 0.0751	6.33 ± 0.56		0.9569 ± 0.0361	4.62 ± 0.54	
	289	0.8965 ± 0.0859	5.58 ± 0.62		0.9296 ± 0.0552	6.07 ± 1.31	
	293	0.9796 ± 0.0134	4.90 ± 0.88		0.9289 ± 0.0479	8.41 ± 1.78	
Nutmeg	277	0.9946 ± 0.0099	1.05 ± 0.50	3.44	0.9992 ± 0.0011	0.44 ± 0.16	3.98
	281	0.6991 ± 0.4739	5.88 ± 0.88		0.9949 ± 0.0062	0.91 ± 0.31	
	289	0.9337 ± 0.0251	6.35 ± 0.11		0.9872 ± 0.0102	2.48 ± 0.32	
	293	0.8144 ± 0.1296	12.43 ± 1.15		0.9991 ± 0.0005	1.19 ± 0.26	
Onion	277	0.9626 ± 0.0331	3.18 ± 0.64	3.68	0.8167 ± 0.1514	5.51 ± 0.98	3.67
	281	0.8429 ± 0.1192	9.79 ± 0.31		0.8732 ± 0.0950	7.23 ± 2.59	
	289	0.9054 ± 0.0780	5.54 ± 0.31		0.9919 ± 0.0073	1.51 ± 0.38	
	293	0.9669 ± 0.0214	6.23 ± 0.61		0.9840 ± 0.0164	2.36 ± 0.80	
Oregano	277	0.8917 ± 0.0961	6.04 ± 1.59	3.73	0.9055 ± 0.0759	5.25 ± 0.65	3.81
	281	0.8952 ± 0.0799	7.69 ± 0.85		0.9111 ± 0.0639	7.23 ± 1.56	
	289	0.9695 ± 0.0281	4.36 ± 1.71		0.9968 ± 0.0025	1.48 ± 0.37	
	293	0.9774 ± 0.0157	5.09 ± 0.14		0.9987 ± 0.0010	1.17 ± 0.15	
Rosemary	277	0.9589 ± 0.0359	2.86 ± 0.38	3.86	0.9805 ± 0.0205	2.79 ± 0.76	3.83
	281	0.9400 ± 0.0504	3.96 ± 0.16		0.9714 ± 0.0232	3.80 ± 0.31	
	289	0.9825 ± 0.0158	2.33 ± 0.42		0.9889 ± 0.0133	2.38 ± 0.88	
	293	0.9807 ± 0.0147	4.22 ± 0.29		0.8927 ± 0.0554	13.96 ± 0.43	
Thyme	277	0.9772 ± 0.0191	2.37 ± 0.08	3.63	0.7280 ± 0.2073	10.27 ± 0.93	3.54
	281	0.8840 ± 0.0953	5.41 ± 0.62		0.9619 ± 0.0252	5.37 ± 0.72	
	289	0.7943 ± 0.1682	9.67 ± 0.71		0.9352 ± 0.0479	6.32 ± 0.13	
	293	0.9794 ± 0.0130	5.54 ± 0.32		0.9125 ± 0.0443	11.38 ± 1.28	

**Table 4:** ANN model parameters for thiol group content in raw and cooked ground chicken meat enriched with plant extracts stored at different temperatures.

Net Parameters	Net Structure				
	MLP 20-14-1	MLP 20-19-1	MLP 20-29-1	MLP 20-28-1	MLP 20-12-1
Training accuracy	0.991	0.994	0.985	0.991	0.991
Test accuracy	0.985	0.987	0.977	0.982	0.985
Validation accuracy	0.984	0.985	0.978	0.982	0.980
Training error	3.913	2.548	6.237	3.766	3.730
Test error	7.398	6.528	10.971	8.473	7.414
Validation error	6.728	6.145	9.140	7.511	8.102
Training algorithm	BFGS 192	BFGS 274	BFGS 275	BFGS 280	BFGS 297
Error function	SOS	SOS	SOS	SOS	SOS
Hidden activation	Tanh	Tanh	Exponential	Logistic	Logistic
Output activation	Tanh	Tanh	Linear	Linear	Tanh
RMSE	2.127	1.884	2.733	2.185	2.367
R^2^	0.979	0.983	0.965	0.977	0.974

**Table 5:** The results of multiple regression analysis.

Independent Variables and Intercept	Slope	
	Raw Meat	Cooked Meat
Allspice	4.78 *	−0.03
Basil	2.87	2.59
Bay leaf	13.48 *	5.59 *
Black seed	9.26 *	−0.97
Caraway	7.70 *	14.30 *
Cardamon	1.61	0.44
Clove	12.26 *	5.31 *
Garlic	6.79 *	−2.03
Nutmeg	7.27 *	8.49 *
Onion	3.06	8.76 *
Oregano	2.48	2.40
Rosemary	8.56 *	−2.61
Thyme	8.20 *	−4.36 *
Temperature	−1.80 *	−1.74 *
Time	−7.89 *	−9.27 *
Intercept	110.76 *	115.03 *

* Paired comparison (control compared with other natural additives) were significantly different to the control at *p* ≤ 0.05.

## Data Availability

The data that support the findings of this study are published in Mendeley datasets at Kaczmarek, Anna (2021), “SH_CHICKEN”, Mendeley Data, V1, doi:10.17632/jnwnvvsvcz.1.

## References

[1] Sobral M.M.C., Casal S., Faria M.A., Cunha S.C., Ferreira I.M.P.L.V.O. (2020). Influence of culinary practices on protein and lipid oxidation of chicken meat burgers during cooking and in vitro gastrointestinal digestion. Food Chem. Toxicol..

[2] Lund M.N., Heinonen M., Baron C.P., Estévez M. (2011). Protein oxidation in muscle foods: A review. Mol. Nutr. Food Res..

[3] Kim Y.H., Huff-Lonergan E., Sebranek J.G., Lonergan S.M. (2010). High-oxygen modified atmosphere packaging system induces lipid and myoglobin oxidation and protein polymerization. Meat Sci..

[4] Huff-Lonergan E., Lonergan S.M. (2005). Mechanisms of water-holding capacity of meat: The role of postmortem biochemical and structural changes. Meat Sci..

[5] Goethals S., Van Hecke T., Vossen E., Vanhaecke L., Van Camp J. (2020). Commercial luncheon meat products and their in vitro gastrointestinal digests contain more protein carbonyl compounds but less lipid oxidation products compared to fresh pork. Food Res. Int..

[6] Estévez M., Luna C. (2017). Dietary protein oxidation: A silent threat to human health?. Crit. Rev. Food Sci. Nutr..

[7] Estévez M., Li Z., Soladoye O.P., Van-Hecke T. (2017). Health Risks of Food Oxidation. Advances in Food and Nutrition Research.

[8] Munekata P.E.S., Rocchetti G., Pateiro M., Lucini L., Domínguez R., Lorenzo J.M. (2020). Addition of plant extracts to meat and meat products to extend shelf-life and health-promoting attributes: An overview. Curr. Opin. Food Sci..

[9] Burri S., Granheimer K., Rémy M., Tannira V., So Y., Rumpunen K., Tornberg E., Canaviri Paz P., Uhlig E., Oscarsson E. (2020). Processed meat products with added plant antioxidants affect the microbiota and immune response in C57BL/6JRj mice with cyclically induced chronic inflammation. Biomed. Pharmacother..

[10] Hellwig M. (2019). The Chemistry of Protein Oxidation in Food. Angew. Chem. Int. Ed..

[11] Jongberg S., Racanicci A.M.C.C., Skibsted L.H. (2019). Mate extract is superior to green tea extract in the protection against chicken meat protein thiol oxidation. Food Chem..

[12] Jongberg S., Tørngren M.A., Gunvig A., Skibsted L.H., Lund M.N. (2012). Effect of green tea or rosemary extract on protein oxidation in Bologna type sausages prepared from oxidatively stressed pork. Meat Sci..

[13] Nieto G., Jongberg S., Andersen M.L., Skibsted L.H. (2013). Thiol oxidation and protein cross-link formation during chill storage of pork patties added essential oil of oregano, rosemary, or garlic. Meat Sci..

[14] Hellwig M. (2019). Die Chemie der Proteinoxidation in Lebensmitteln. Angew. Chem..

[15] Silva F.A., Estévez M., Ferreira V.C., Silva S.A., Lemos L.T., Ida E.I., Shimokomaki M., Madruga M.S. (2018). Protein and lipid oxidations in jerky chicken and consequences on sensory quality. LWT.

[16] Rysman T., Van Hecke T., Van Poucke C., De Smet S., Van Royen G. (2016). Protein oxidation and proteolysis during storage and in vitro digestion of pork and beef patties. Food Chem..

[17] Lund M.N., Lametsch R., Hviid M.S., Jensen O.N., Skibsted L.H. (2007). High-oxygen packaging atmosphere influences protein oxidation and tenderness of porcine longissimus dorsi during chill storage. Meat Sci..

[18] Zainudin M.A.M., Jongberg S., Lund M.N., Asraf M., Zainudin M.A.M., Jongberg S., Lund M.N. (2021). Combination of light and oxygen accelerates formation of covalent protein-polyphenol bonding during chill storage of meat added 4-methyl catechol. Food Chem..

[19] Tuell J.R., Park J.-Y., Wang W., Cheng H.-W., Kim Y.H.B. (2020). Functional/physicochemical properties and oxidative stability of ground meat from broilers reared under different photoperiods. Poult. Sci..

[20] Silva A.A., De Melo M.P., Silva S.L., Lins P.G., Lobo A.R., Fernandes R.P.P., Amaral N.R., Taniguchi M.V., Lopes N.P. Protein oxidation and color stability in meat aged under aerobic conditions from bull and steer. Proceedings of the 58th International Congress of Meat Science and Technology.

[21] Weiss D., Kaczmarek A., Stangierski J. (2015). Applicability of bacterial growth models in spreadable processed cheese. Acta Sci. Pol. Technol. Aliment..

[22] Stangierski J., Weiss D., Kaczmarek A. (2019). Multiple regression models and Artificial Neural Network (ANN) as prediction tools of changes in overall quality during the storage of spreadable processed Gouda cheese. Eur. Food Res. Technol..

[23] Limbo S., Torri L., Sinelli N., Franzetti L., Casiraghi E. (2010). Evaluation and predictive modeling of shelf life of minced beef stored in high-oxygen modified atmosphere packaging at different temperatures. Meat Sci..

[24] Wang H., Kong C., Li D., Qin N., Fan H. (2015). Modeling Quality Changes in Brined Bream (*Megalobrama amblycephala*) Fillets During Storage: Comparison of the Arrhenius Model, BP, and RBF Neural Network. Food Bioprocess Technol..

[25] Wenjiao F., Yongkui Z., Yunchuan C., Junxiu S., Yuwen Y. (2014). TBARS predictive models of pork sausages stored at different temperatures. Meat Sci..

[26] Bao Y., Zhou Z., Lu H., Luo Y., Shen H. (2013). Modelling quality changes in Songpu mirror carp (*Cyprinus carpio*) fillets stored at chilled temperatures: Comparison between Arrhenius model and log-logistic model. Int. J. Food Sci. Technol..

[27] Guo Z., Ge X., Yu Q.L., Han L., Zhao H., Cao H. (2018). Quality predictive models for bovine liver during storage and changes in volatile flavors. Int. J. Food Prop..

[28] Delgado A., Rauh C., Park J., Kim Y., Groß F., Diez L. (2016). Artificial Neural Networks: Applications in Food Processing. Reference Module in Food Science.

[29] Panagou E.Z., Mohareb F.R., Argyri A.A., Bessant C.M., Nychas G.J.E. (2011). A comparison of artificial neural networks and partial least squares modelling for the rapid detection of the microbial spoilage of beef fillets based on Fourier transform infrared spectral fingerprints. Food Microbiol..

[30] Singh R., Ruhil A., Jain D., Patel A., Patil G. (2009). Prediction of sensory quality of UHT milk—A comparison of kinetic and neural network approaches. J. Food Eng..

[31] Muzolf-Panek M., Kaczmarek A., Tomaszewska-Gras J., Cegielska-Radziejewska R., Szablewski T., Majcher M., Stuper-Szablewska K. (2020). A Chemometric Approach to Oxidative Stability and Physicochemical Quality of Raw Ground Chicken Meat Affected by Black Seed and Other Spice Extracts. Antioxidants.

[32] Sánchez-Moreno C., Larrauri J.A., Saura-Calixto F. (1998). A procedure to measure the antiradical efficiency of polyphenols. J. Sci. Food Agric..

[33] Muzolf-Panek M., Kaczmarek A., Tomaszewska-Gras J., Cegielska-Radziejewska R., Majcher M. (2019). Oxidative and microbiological stability of raw ground pork during chilled storage as affected by Plant extracts. Int. J. Food Prop..

[34] Singleton V.L., Rossi J.A. (1965). Colorimetry of Total Phenolics with Phosphomolybdic-Phosphotungstic Acid Reagents. Am. J. Enol. Vitic..

[35] Kaczmarek A., Muzolf-Panek M. (2021). Predictive Modeling of Changes in TBARS in the Intramuscular Lipid Fraction of Raw Ground Beef Enriched with Plant Extracts. Antioxidants.

[36] Van Boekel M.A.J.S. (2008). Kinetic modeling of food quality: A critical review. Compr. Rev. Food Sci. Food Saf..

[37] Ratkowsky D.A., Olley J., Mcmeekin T.A., Ball A. (1982). Relationship Between Temperature and Growth Rate of Bacterial Cultures. J. Bacteriol..

[38] Van Boekel M.A.J.S. (2009). Kinetic Modeling of Reactions in Foods.

[39] Guyon C., Meynier A., de Lamballerie M. (2016). Protein and lipid oxidation in meat: A review with emphasis on high-pressure treatments. Trends Food Sci. Technol..

[40] Wang Z.Z., He Z., Zhang D., Li H., Wang Z.Z. (2020). Using oxidation kinetic models to predict the quality indices of rabbit meat under different storage temperatures. Meat Sci..

[41] Radha Krishnan K., Babuskin S., Azhagu Saravana Babu P., Sivarajan M., Sukumar M. (2015). Evaluation and predictive modeling the effects of spice extracts on raw chicken meat stored at different temperatures. J. Food Eng..

[42] Olivera D.F., Bambicha R., Laporte G., Cárdenas F.C., Mestorino N. (2013). Kinetics of colour and texture changes of beef during storage. J. Food Sci. Technol..

[43] Kaczmarek A., Cegielska-Radziejewska R., Szablewski T., Zabielski J. (2015). TBARS and microbial growth predicative models of pork sausage stored at different temperatures. Czech, J. Food Sci..

[44] Xu Z., Liu X., Wang H., Hong H., Luo Y. (2017). Comparison between the Arrhenius model and the radial basis function neural network (RBFNN) model for predicting quality changes of frozen shrimp (*Solenocera melantho*). Int. J. Food Prop..

[45] Tsironi T., Salapa I., Taoukis P. (2009). Shelf life modelling of osmotically treated chilled gilthead seabream fillets. Innov. Food Sci. Emerg. Technol..

[46] Gallo M., Ferracane R., Naviglio D. (2012). Antioxidant addition to prevent lipid and protein oxidation in chicken meat mixed with supercritical extracts of Echinacea angustifolia. J. Supercrit. Fluids.

